# Comprehensive Characterization of Human Genome Variation by High Coverage Whole-Genome Sequencing of Forty Four Caucasians

**DOI:** 10.1371/journal.pone.0059494

**Published:** 2013-04-05

**Authors:** Hui Shen, Jian Li, Jigang Zhang, Chao Xu, Yan Jiang, Zikai Wu, Fuping Zhao, Li Liao, Jun Chen, Yong Lin, Qing Tian, Christopher J. Papasian, Hong-Wen Deng

**Affiliations:** 1 Center for Bioinformatics and Genomics, Department of Biostatistics and Bioinformatics, School of Public Health and Tropical Medicine, Tulane University, New Orleans, Louisiana, United States of America; 2 School of Medicine, University of Missouri-Kansas City, Kansas City, Missouri, United States of America; 3 Center of System Biomedical Sciences, University of Shanghai for Science and Technology, Shanghai, P. R. China; University of Montreal, Canada

## Abstract

Whole genome sequencing studies are essential to obtain a comprehensive understanding of the vast pattern of human genomic variations. Here we report the results of a high-coverage whole genome sequencing study for 44 unrelated healthy Caucasian adults, each sequenced to over 50-fold coverage (averaging 65.8×). We identified approximately 11 million single nucleotide polymorphisms (SNPs), 2.8 million short insertions and deletions, and over 500,000 block substitutions. We showed that, although previous studies, including the 1000 Genomes Project Phase 1 study, have catalogued the vast majority of common SNPs, many of the low-frequency and rare variants remain undiscovered. For instance, approximately 1.4 million SNPs and 1.3 million short indels that we found were novel to both the dbSNP and the 1000 Genomes Project Phase 1 data sets, and the majority of which (∼96%) have a minor allele frequency less than 5%. On average, each individual genome carried ∼3.3 million SNPs and ∼492,000 indels/block substitutions, including approximately 179 variants that were predicted to cause loss of function of the gene products. Moreover, each individual genome carried an average of 44 such loss-of-function variants in a homozygous state, which would completely “knock out” the corresponding genes. Across all the 44 genomes, a total of 182 genes were “knocked-out” in at least one individual genome, among which 46 genes were “knocked out” in over 30% of our samples, suggesting that a number of genes are commonly “knocked-out” in general populations. Gene ontology analysis suggested that these commonly “knocked-out” genes are enriched in biological process related to antigen processing and immune response. Our results contribute towards a comprehensive characterization of human genomic variation, especially for less-common and rare variants, and provide an invaluable resource for future genetic studies of human variation and diseases.

## Introduction

Genome-wide association studies (GWAS) have identified a large number of genetic variants that are associated with a variety of human complex diseases/traits [1], [2]. A major challenge in this post-GWAS era is to pinpoint the functional variants underlying the observed associations and to identify the missing heritability [3]–[6], which requires a comprehensive identification and characterization of genetic variants in the human genome. The rapidly evolving massively-parallel DNA sequencing technology enables efficient and cost-effective whole genome sequencing [7]–[10], and is revolutionizing our understanding of the human genome architecture and variation, human evolution, and the genomics of common and rare disorders [11]–[26]. The 1000 Genomes Project Consortium recently reported results for the Phase 1 of the project [12], [20]. By performing mainly low-coverage whole genome sequencing and exon-targeted sequencing, the consortium identified approximately 15 million single nucleotide polymorphisms (SNPs), 1 million short insertions and deletions (indels), and over 20,000 structural variants [12], [20]. More recently, a few high-coverage sequencing studies have been carried out at whole genome level [14] or at target genes [27], [28] and discovered a large number of previously unidentified variants, suggesting that a considerable number of human genetic variants, particularly rare variants, remain to be discovered beyond those currently archived in the dbSNP and the 1000 Genomes Project.

These initial studies attest to the necessity for performing whole genome sequencing analysis on additional human samples, particularly at high-coverage, in order to gain a comprehensive understanding of the human genomic variation. Here we report the results of a whole genome sequencing study for 44 Caucasian subjects from a single population in Midwest USA, all of whom were sequenced at high coverage. This study represents the first few high-coverage analyses of multiple genomes for healthy human subjects in a single population of the same ethnicity. Our results contribute towards a more comprehensive characterization of human genomic variation, especially for less-common and rare variants, and provide a valuable resource for future genetic studies of human variation and diseases.

## Results

### Sequence data generation and mapping

Genomic DNA samples from 44 healthy, self-reported US Caucasian adults from Midwest USA (in Kansas City and its vicinities), including 22 males and 22 females, were sequenced at Complete Genomics Inc. (Mountain View, CA). All participants signed an informed-consent document before entering the study.

For each individual, 147.7–229.6 gigabases (Gb) of sequence were generated and mapped to the NCBI human reference genome (build 37.1, GRCh37/hg19), resulting in an average of 65.8-fold (range: 51.7–80.3×) genomic coverage (Table 1 and Table S1), which is significantly higher than other reported population-based whole genome sequencing studies. Diploid calls were confidently made for an average 96.0% of the autosomal bases in the reference genome, with a range of 93.8% to 96.9% across the 44 genomes (Table 1 and Table S1).

**Table 1:** Summary information of population-based whole-genome sequencing studies.

	Aligned Bases (Gb)	Coverage depth	Genome Covered	SNPs (Total)	Indels/Subs (Total)
Current study (N = 44)	188.08	65.8×	96.0%	3,307,678 (10,871,465)	492,486 (3,209,732)
Korean WGS (N = 10)	74.09	26.1×	NA	3,602,372 (8,367,302)	332,561 (1,191,599)
Duke WGS^1^ (N = 20)	NA	31.1×	97.5%	3,473,639 (10,530,094)	609,795 (2,736,907)
1000 G_LC^2^ (N = 179)	10.51	3.56×	86.0%	3,019,919 (14,894,361)	361,669 (1,330,158)
1000 G_HCT^3^ (N = 6)	118.5	41.6×	79.0%	3,001,156 (5,907,699)	352,474 (682,148)

Numbers shown are average numbers per individual except where indicated otherwise.

1 For autosomes only; ^2^ 1000 G_LC: Low-coverage samples from the 1000 Genomes Project; ^3^ 1000 G_HCT: High-coverage trios from the 1000 Genomes Project.

### SNP and indel identification and characterization

In total, 10,871,465 distinct SNPs were identified in the 44 genomes, with an average of 3.3 million SNPs per genome (Table 1). The average SNP transition to transversion ratio was 2.13, and the average SNP heterozygote to homozygote ratio was 1.56 (Table S1), both consistent with previous reports [7], [9], [21]. In addition, we identified a total of 1,350,484 distinct short insertions (range 1–76 bp) and 1,464,731 distinct short deletions (range 1–192 bp) in our sample, with an average of 207,000 short insertions and 214,000 short deletions in an individual genome (Table 2, Table S1). The estimated heterozygote to homozygote ratios were 1.4–1.7 for short indels (Table 2, Table S1), also comparable to those calculated from a previous study [11]. Multiple SNPs and/or indels that are in close proximity (i.e., less than two reference bases between two variant sequences) were grouped together as a single variant locus, termed block substitutions. In total, we identified 394,517 distinct block substitutions in our sample, with each individual genome carrying approximately 71,398 such block substitutions (Table 2, Table S1). These block substitutions can be length-conversing (the sample's allele and reference are the same length) or length-changing, and the change in sequence length caused by these substitutions ranges 0–183 bp (Figure S3).

**Table 2:** Summary of identified SNPs and indels/block substitutions.

Variant Type	Total No. of Variants	Average No. of Variants in individual genomes
SNP	10,871,465	3,307,678
Intergenic	6,674,155	2,054,900
Intragenic	4,197,310	1,252,778
Intron	3,473,672	1,043,427
UTR	614,753	181,267
Splicing acceptor site	6,644	1,898
Splicing donor site	1,547	398
Coding domain	79,762	18,796
Synonymous	36,538	9,612
Non-synonymous	43,224	9,184
Missense	42,549	9,082
Nonsense	616	87
Nonstop	59	15
Indels	2,815,215	421,088
Coding domain	3,606	381
Frameshift	2,344	217
Frameshift-preserving	1,262	164
Block substitutions	394,517	71,398
Coding domain	1,870	274
Synonymous	30	20
Frameshift	264	17
Missense	1,546	234
Nonsense	29	3
Nonstop	1	<1

**Figure 1:**
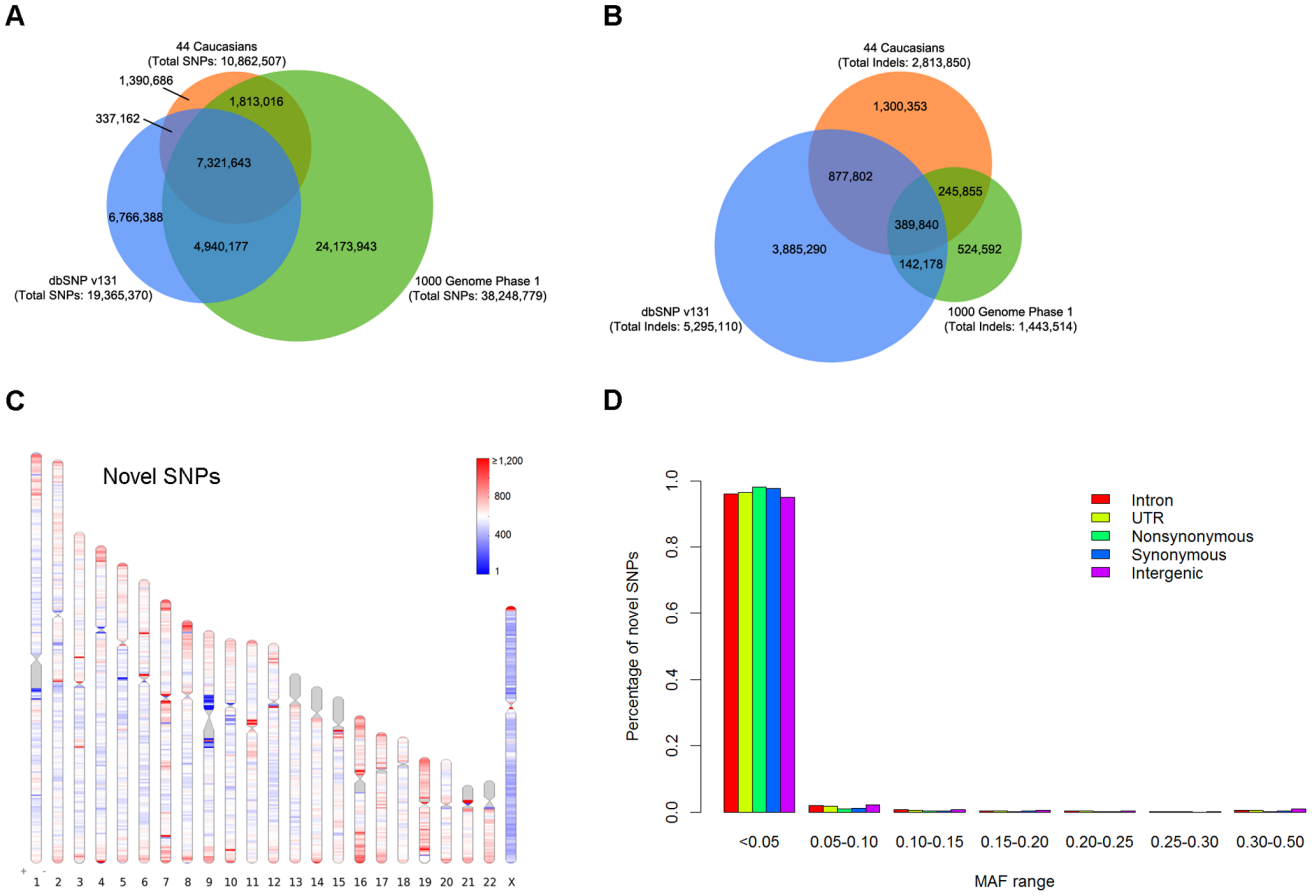
Summary characterizations of the identified variants. **A–B,** Venn diagram showing SNPs and indels identified in the present study overlapping with those archived in the dbSNP (v131) and the 1000 Genomes Project Phase 1 data sets (released on 5/21/2011). To account for differences in placement of many indels between different data sets, indels were considered to match if they were within 25 bp distance and of the same size. Only SNPs and indels mapped to autosomes and X chromosome were analyzed. **C,** Genome-wide distribution of novel SNPs. Total number of novel SNPs (compared to dbSNP v131 and the 1000 Genomes Project pilot phase) were calculated in non-overlap 1-megabases (Mb) windows across the human genome and plotted in ideograms using *Idiographica* [41]. The diversities were illustrated by colors, with red indicating higher numbers or proportions and blue indicating lower numbers or proportions. Genomic regions in which no SNPs were identified or no reference sequences could be determined are shown in grey. **D,** Allele frequency spectrum of novel SNPs.

Of all the SNPs (n = 10,862,507) mapped to autosomes and X chromosome, 7,658,805 (70.5%) were already present in dbSNP (v131), and 9,134,659 (84.1%) were in the 1000 Genomes Project Phase 1 data set (released on 5/21/2011, http://www.1000genomes.org/), whereas the rest 1,390,686 (12.8%) SNPs were in neither dataset and thus considered to be novel (Fig. 1a). In contrast, a large proportion (46.2%) of the identified short indels was novel relative to dbSNP (v131) and the 1000 Genomes Project Phase 1 data set (Fig. 1b). Interestingly, short indels reported in any of the three data sets were largely unique to that data set, showing only ∼20–40% overlap with any other data set (Fig. 1b). The limited overlap of indels identified between different data sets or between different individual genomes has been previously recognized [11], [12], [15], [25]. This observation may reflect the substantial challenges in making accurate calls of indels. For instance, the estimated false discovery rates for indels were fairly high by both the Complete Genomics approach (∼3.0–6.5%) [8] and the 1000 Genomes project approach (∼3.7–8.1%) [29]. Moreover, 18% of indel sites identified by the 1000 Genomes project were found to be inconsistent or ambiguous [29].These results highlighted the need for developing advanced experimental and analytic methods for identification and characterization of indels.

Consistent with previous reports [12], [13], high densities of variations were observed at the human leukocyte antigen (HLA) region, and a number of telomeric and sub-telomeric regions (Fig. S1). In general, we observed strong correlations in genomic distributions between distinct forms of variants (Fig. S2). Specifically, short insertions and deletions showed very strong correlation throughout the entire genome, whereas block substitutions were relatively poorly correlated with other forms of variants (Fig. S2).

The genome-wide distribution of novel SNPs (Fig. 1c) exhibited similar patterns as that of the total SNPs (Fig. S1). As expected, the vast majority (∼96%) of novel SNPs represented low-frequency (minor allele frequency, MAF, ≤5%) variants (Fig. 1d), as most common SNPs in human populations were already included in dbSNP. Intriguingly, several regions contained relatively high proportions of novel SNPs (Fig. S1). For instance, 75–83% of all SNPs identified at centromeric regions on chromosomes 3, 7, 16 and X, were not in the dbSNP (Table S2). In addition, similarly high fractions of novel SNPs were also observed in these regions, when comparing our data with the 1000 Genomes Project Phase 1 data set (novel rate: 80–97%) and when comparing the 1000 Genomes Project Phase 1 data set with the dbSNP (novel rate: 84–91%) (Table S2). The high discrepancy between SNPs from different data sets that are mapped to these regions may suggest that it is difficult to accurately sequence these regions with currently available sequencing techniques.

To evaluate the accuracy of our data, we first estimated the SNP genotyping concordance between whole genome sequencing and SNP arrays for 20 subjects that had been genotyped with the Affymetrix Genome-Wide Human SNP Array 6.0. Out of the 906,600 SNPs called on the SNP arrays, 801,557 (98.5%) were successfully called by the present sequencing project and the genotype concordance rates were 99.83% and 99.45% for homozygous and heterozygous SNPs, respectively. By comparing with the recently reported 10 Korean genomes [14], the 69 genomes data set from Complete Genomics Inc (http://www.completegenomics.com/sequence-data/download-data/), and an exome sequencing data of 100 Han Chinese subjects (Lin et al., unpublished), we *in silico* validated 177,554 SNPs and 411,658 indels that were not archived in the dbSNP (v131) or the 1000 Genomes Project Phase 1 data sets, supporting that these novel variants are likely to be genuine polymorphisms and not just unique to our specific population. To further evaluate the accuracy of the novel variants, we tested 25 novel nonsynonymous SNPs by traditional Sanger sequencing. Twenty-four SNPs out of the 25 were validated, highlighting the high quality and reproducibility of our data.

### Gene-based characterization of SNPs and indels

Of all the identified SNPs, 79,762 were mapped to CDS (coding sequences) of RefSeq transcripts, including 36,538 synonymous, 42,549 missense, 616 nonsense, and 59 nonstop (causing the loss of a stop codon) SNPs (Table 2). Consistent with previous reports [12], [13], we observed a significantly higher fraction of low-frequency variants (MAF≤0.05) in non-synonymous SNPs, when compared to synonymous or noncoding SNPs, presumably reflecting purifying selection against deleterious mutations (Fig. S3). Of all the identified indels, only a very small fraction (0.13%) is mapped to coding sequence regions and most of the coding indels were predicted to cause frameshift changes (Table 2), which is consistent with previous reports [12], [13]. We observed a noticeable preference for 3n-bp indels in coding regions compared to indels in non-coding genic regions, such as in introns and UTRs (Fig. S3). This preference has also been observed in other studies [9], [13], [30] and may reflect the selection pressure for minimally impacting variants in coding regions. In addition, we detected 1,870 block substitutions that are mapped to coding sequence regions, and almost all of the coding block substitutions were predicted to cause non-synonymous changes (Table 2). This is expected, as block substitutions involve multiple variant bases and thus are more likely to result in amino acid changes. Similar bias towards non-synonymous variants were also reported in a previous study by Rosenfeld et al [31], who focused on a specific type of block substitution, namely, double nucleotide polymorphisms (DNPs) and triple nucleotide polymorphisms (TNPs), which by definition are length-conserving block substitutions involving two or three consecutive nucleotides.

We performed GO (Gene Ontology) analyses to determine whether genes containing high density of amino acid changing variants were enriched in specific biological processes. The GO terms related to sensory perception, such as ‘sensory perception of chemical stimulus’ (p = 3.47×10^−8^) and ‘sensory perception of smell’ (p = 7.21×10^−7^) showed significant enrichment for genes with high density of amino acid changing variants (Table 3). In contrast, amino acid changing variants were significantly under-represented in multiple biological processes related to ‘cellular macromolecule metabolic process’ (p = 1.38×10^−19^) and ‘RNA metabolic process’ (p = 1.52×10^−17^) (Table 3). These results were not unexpected because these metabolic and signal transduction related biological processes are critical for cell survival and functioning, consequently, are expected to be under strong purifying selection against deleterious mutations. On the other hand, sensory perception related genes are known to be highly associated with environmental adaptation. Thus, variants may have accumulated that differ in frequencies among human populations, suggesting balanced selection, the possible relaxation of purifying selection, and/or an increased mutation rate [9], [15].

**Table 3:** Top 10 GO terms significantly enriched or depleted for deleterious coding variants, and enriched for “knocked-out” genes.

GO Accession #		Biological Process	P-value
*Enriched for deleterious coding variants*			
GO:0050907		detection of chemical stimulus involved in sensory perception	2.97E–09
GO:0007606		sensory perception of chemical stimulus	3.47E–08
GO:0050911		detection of chemical stimulus involved in sensory perception of smell	1.03E–07
GO:0009593		detection of chemical stimulus	3.22E–07
GO:0007608		sensory perception of smell	7.21E–07
*Depleted for amino acid changing variants*			
GO:0044260		cellular macromolecule metabolic process	1.38E–19
GO:0009987		cellular process	1.31E–18
GO:0016070		RNA metabolic process	1.52E–17
GO:0043170		macromolecule metabolic process	1.96E–15
GO:0006139		nucleobase-containing compound metabolic process	2.04E–15
*Enriched for “knocked-out” gene*			
GO:0002474	antigen processing and presentation of peptide antigen via MHC class I		1.79E–23
GO:0019882	antigen processing and presentation		1.18E–21
GO:0048002	antigen processing and presentation of peptide antigen		1.65E–18
GO:0006611	protein export from nucleus		2.95E–12
GO:0006955	immune responses		3.56E–08

P-values were computed for significance of enrichment by *Gorilla (* http://cbl-gorilla.cs.technion.ac.il/ *)*.

**Figure 2:**
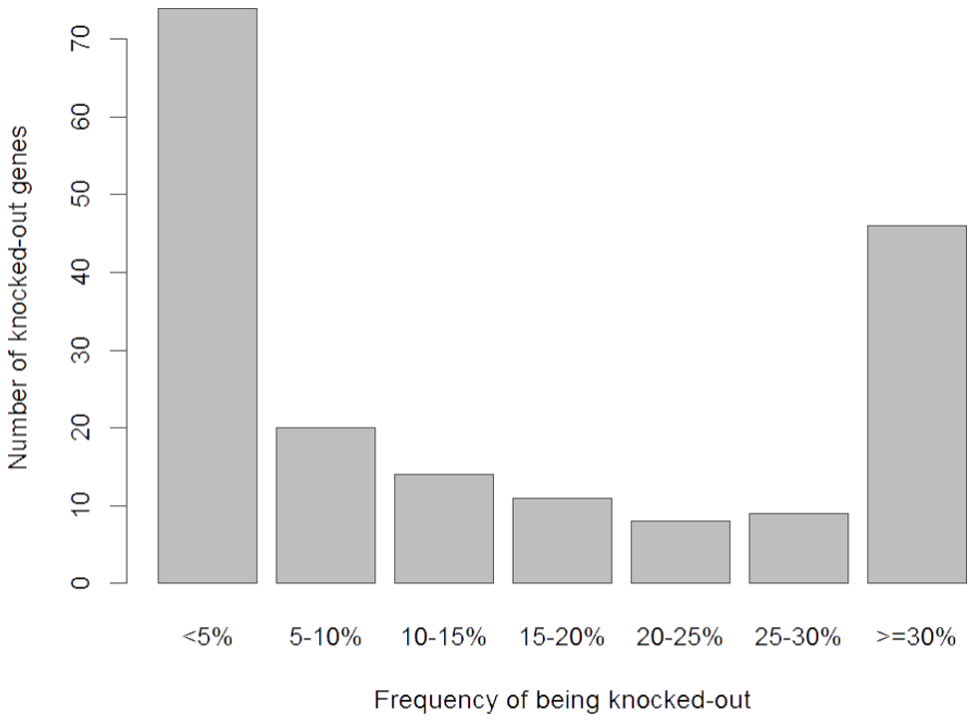
Identification of “knocked-out” genes. **A,** Frequency spectrum of observed “knocked-out” genes. Genes containing homozygous LoF variants were expected to be silent or knocked-out. Numbers of “knocked-out” genes were counted with respect to the frequency of “knock-out” occurrence in the 44 genomes.

Using the Variant Annotation Tool (version 2.0.1.) [32] with the GENCODE v7 annotation [33], we examined SNPs and indels for variants predicted to result in the complete loss-of-function (LoF). LoF variants were defined as SNPs predicted to create or disrupt a stop codon, frameshift indels, and variants predicted to disrupt a splice site. On average, each person carried approximately 179 LoF variants with ∼44 of these variants occurred in a homozygous state, which would result in complete “knock-out” of the annotated genes. Altogether, we found 182 unique “knocked-out” genes in our samples. While about 40% of these genes were “knocked out” in just one or two individuals, 46 genes were “knocked out” in over 30% of our samples (Fig. 2), suggesting that a number of genes are commonly “knocked-out” in general populations. GO analysis suggested that these “knocked-out” genes were significantly enriched in several biological processes, particularly those related to antigen processing and immune responses (Table 3). Intriguingly, 8 LoF variants located in 8 genes (Table S3) were found to be homozygous in all the 44 genomes, as well as in other independently sequenced human genomes [26], [30], suggesting that the reference sequences at these sites may represent rare alleles or sequencing errors.

### Disease-associated SNPs and indels

Using *Trait-o-matic* (https://github.com/xwu/trait-o-matic), we found that each individual genome carried an average of 90 non-synonymous SNPs that were known to be associated with OMIM diseases/traits. Among the 44 samples, 29 subjects carried 14 heterozygous variants causing autosomal dominant diseases/traits, and 4 subjects were homozygous for 4 variants causing autosomal recessive diseases/traits (Table S4). However, the 44 subjects were all apparently healthy with no recognizable or self-reported diseases at the time of enrollment. This might be explained by the fact that the majority of these variants were known to have reduced/age-dependent penetrance (e.g., R468H mutation in *MFN2* gene for Charcot-Marie-Tooth disease-2A2) and/or did not cause severe health-threatening symptoms (e.g., wet/dry ear wax). This perspective is supported by the fact that 8 of the 18 variants were present in low to intermediate frequency in general populations (Table S4). Alternatively, some of these variants might have been erroneously assigned as disease mutations. Each genome also contained approximately 276 additional non-synonymous SNPs, including about 34 potential nonsense/nonstop SNPs and 11 frameshift indels and block substitutions in genes associated with OMIM diseases/traits.

### Mitochondrial and Y chromosome analyses

By comparing to the revised Cambridge Reference Sequence (rCRS) of the Human Mitochondrial DNA [34], we identified a total of 306 mitochondrial variants, including 285 SNPs, 13 short indels, and 8 block substitutions (Table S5). Approximately 54% of these SNPs, and almost all the indels and block substitutions, were novel relative to dbSNP (v131). In the 37 mitochondrial genes, we identified 54 non-synonymous SNPs (53 missense and 1 nonstop), and one frameshift block substitution.

A total of 8,673 SNPs, 1,352 indels, and 998 block substitutions were identified on the Y chromosome (Table S5). Similar to the mitochondrial genome, approximately 54% of these SNPs and the majority (∼65%) of indels/block substitutions on the Y chromosome were novel relative to dbSNP (Table S5).

### Copy number variants (CNVs)

Through the analysis of read depth in 2-kb sliding windows, we discovered an average of 3,749 CNVs (size range of 2–154 kb) totaling about 12 Mb in each genome. Approximately 45% of the CNVs detected in our study overlapped (≥50% of sequence overlap) with CNVs currently annotated in the Database of Genomic Variants (DGV) [35]. Similar to previous reports [9], [20], we observed a steadily decreasing number of events with increasing CNV length (Fig. S4). Out of the 493 CNVs identified in the 20 subjects with the Affymetrix Genome-Wide Human SNP Array 6.0, 255 overlapped with CNVs called by the sequencing data.

### Rates of novel variants discovery

**Figure 3:**
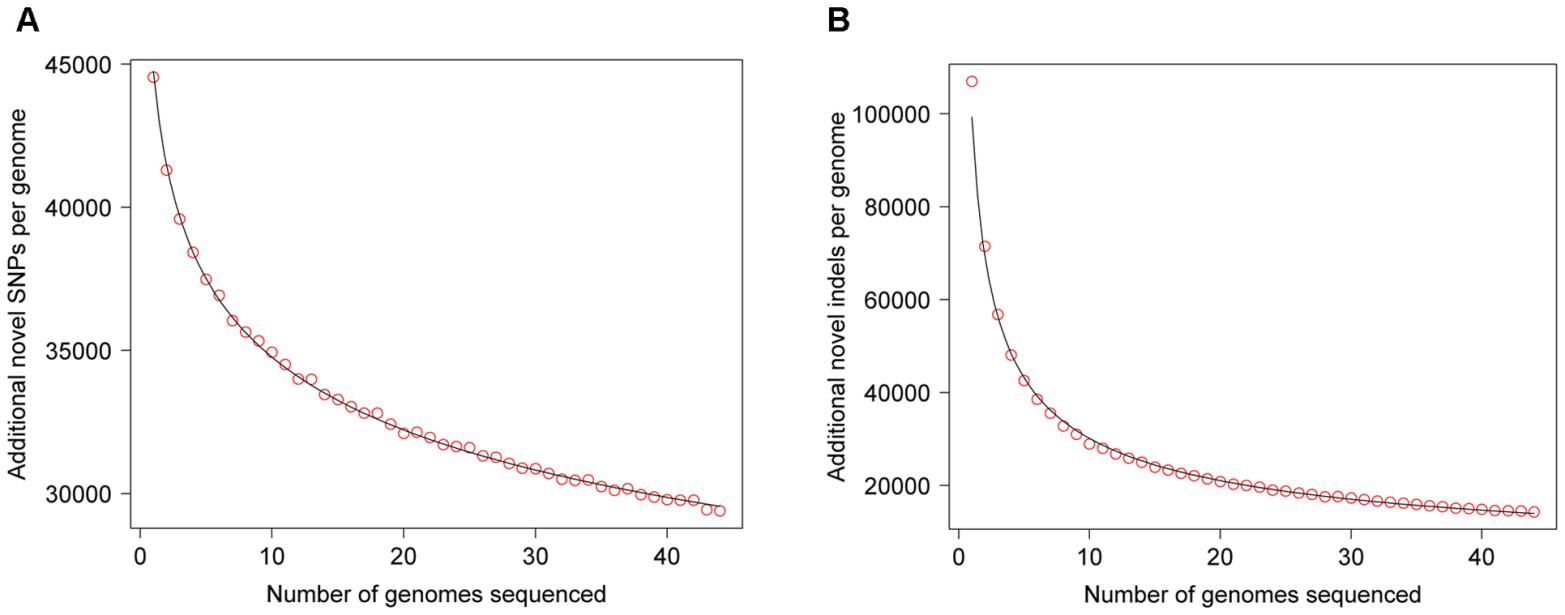
The number of novel SNPs and indels discovered as the number of sequenced genomes increased. We evaluated how many additional “new” **A**) SNPs and **B**) indels, respectively, were identified per genome as the number of sequenced genomes increased, considering both variants archived in databases (dbSNP v131 and the 1000 Genome Project Phase 1 data) and variants “discovered” in previously considered genomes. The 44 genomes were added into the analyses in a random order. With 1000 permutations, the average numbers of novel variants added per genome are shown, along with the best fitting trendline for each plot.

To evaluate how the rate of novel SNP/indel identification changed as the number of sequenced genomes increased, we adopted the permutation method used by Pelak et al. [21]. Briefly, we permuated the order of the 44 personal genomes 1000 times and determined the mean number of “new” variants added by each additional personal genome, simultaneously considering variants archived in dbSNP/1000 Genome Project Phase 1 data sets as well as those discovered in the previously considered genomes. As shown in Figure 3, one randomly selected individual genome contained an average of 44,500 novel SNPs and ∼107,000 novel indels (not in dbSNP/1000 Genome Project Phase 1 data sets). As additional individuals were sequenced, the number of additional novel SNPs and indels (not in dbSNP/1000 Genome Project Phase 1 data sets and the previously sequenced genomes) added per genome gradually declined. After about 40 genomes were sequenced, the discovery rate of additional novel variants appeared to plateau at approximately 29,500 SNPs and 14,300 indels per genome.

## Discussion

Over the past several years, GWAS assaying several hundred thousand to a few million SNPs in thousands of individuals have successfully identified numerous genetic variants that were significantly associated with over two hundred human complex diseases/traits [1], [2]. However, most of these variants are likely to be genetic ‘markers’, rather than actual causative variants, and the vast majority of these variants, individually or in combination, can explain only a small proportion (general <10%) of the heritability for these diseases/traits [5], [6]. The missing heritability is partially attributable to the imprecise estimation of genetic effects based on the disease/trait-associated markers, and rare/low-frequency variants, especially variants other than SNPs, which are poorly covered by current GWAS approaches [5], [6], [36]. In this context, performing an exhaustive inspection of all genetic variants located in the associated regions is essential to pinpoint causative variants and to generate deeper insights into genetic and functional mechanisms underlying the observed associations. Exhaustive inspection of all genetic variants requires a more comprehensive description and understanding of genetic variants in the human genome. Recent advances in DNA sequencing technology have greatly facilitated sequencing of individual genomes. In the present study, we carried out high-coverage (>50×), whole genome sequencing for 44 apparently healthy Caucasians, and identified approximately 11 million SNPs and 3.3 million short indels and block substitutions, including ∼1.4 million SNPs and ∼1.3 million indels that were not cataloged in the dbSNP (v131) and the 1000 Genome Project Phase 1 data set.

The recently reported pilot phase of the 1000 Genomes Project identified a massive number of SNPs and estimated that approximately 95% of all accessible common SNPs were catalogued in their data set [12], [20]. By comparing the SNPs identified in the current study with the 1000 Genomes Project Phase 1 and the dbSNP (v131) data sets, we showed that an average of 95.6% of the SNPs in any individual genome was already reported (Table S1). Our study also identified 1,390,686 novel SNPs and the majority of which (∼96%) were with low and rare frequencies (MAF ≤5%), highlighting the power of rare variant identification by our high-coverage genome sequencing strategy. Based on our evaluation of the discovery rate of novel variants, we can obtain a reasonable estimation of the novel variants contained in a single individual genome. For instance, a single individual genome in our sample contained an average of ∼44,500 SNPs and 107,000 indels that were not archived in the dbSNP (v131) or the 1000 Genome Project Phase 1 data, many of which were expected to be at low frequencies. This number should be interpreted cautiously, particularly for the novel indels, considering the relatively high false discovery rate (3.0–6.5%) [8] in indel identification. On the other hand, given the fact that potential deleterious genetic variants were enriched in the low and rare frequency spectrum, our data suggested that a considerable number of genuine variants that are related to disease susceptibility may still be undiscovered. Consequently, it is critical that additional high-coverage sequencing studies be conducted in additional subjects in order to identify these variants.

Interestingly, we showed that each individual genome from the general population carries approximately 179 potential LoF variants with ∼44 genes expected to be completely disrupted (“knocked-out”) due to LoF variants in homozygous state. Though several earlier studies suggested individual genome may carry 250–300 [12], [18] and perhaps even more [21] potential LoF variants, these numbers should be interpreted cautiously because LoF variants were expected to be highly enriched for false positives [37]. A recent study [38] suggested that many of these previously identified putative LoF variants may represent a variety of sequencing and annotation errors. After applying a series of stringent filters to the putative LoF variants, it was estimated that human genome typically contain ∼100 genuine LoF variants with ∼20 genes completed inactivated [38]. These high-confidence LoF variants have important implications for the interpretation of clinical sequencing studies. On the other hand, these stringent filters were also expected to remove a significant number of true positive LoF variants as well and thus the number of these high-confidence LoF variants may be regarded as a lower bound for the number of LoF variants carried by individual genome [38]. Therefore, our results and similar numbers (∼150 LoF variants per individual genome) recently reported by the 100 Genome Project Consortium [29] may represent a reasonable estimation of the true number of LoF variants and “knocked-out” genes per individual genome. In total, we identified 182 “knocked-out” genes in our samples. The GO term analysis suggested that these “knocked-out” events were more frequently occurred in genes participating in antigen processing and presentation and immune responses, probably reflecting the fact that many genes in the immune systems have redundant biological functions [35]. As more individual genomes being sequenced in the future, we expect to slowly identify many more human genes that are not essential for survival and can be “knocked-out” in the general population. Detailed clinical phenotyping and biological characterization at tissue/cell-level in these individuals will shed light on the functional mechanisms and impacts of these specific “nonessential” genes.

Complete identification and characterization of human DNA variants is essential to further decipher the genetic basis of human evolution and disease susceptibility, and for a comprehensive understanding of human biology. This study, along with previously reported whole genome sequencing projects represents initial, but significant, steps towards a better understanding of the vast human genomic variation.

## Materials and Methods

### Study population

Forty four unrelated healthy Caucasian adults, including twenty two females and twenty two males, were recruited through the Kansas City Osteoporosis Study (KCOS), a genetic repertoire of ∼6,000 subjects collected for genomic studies of complex diseases/traits. All subjects were living in Kansas City, Missouri and its surrounding areas and were self-identified as being of European origin. The study was approved by the University of Missouri-Kansas City (UMKC) Institutional Review Board, and each participant signed an informed-consent document before entering the study.

### Genomic DNA preparation and sequencing

Genomic DNA was isolated from peripheral blood using the Gentra Puregene Blood kit (Qiagen, Valencia, CA) according to the recommended protocol. DNA concentration was measured by Nanodrop 1000 (Thermo Scientific, Wilmington, DE) and Quant-iT*™* Pico Green dsDNA kit (Invitrogen, Carlsbad, CA). Whole genome DNA sequencing was performed by Complete Genomics, Inc (Mountain View, CA), using its paired end library preparation and sequencing-by-ligation methodology as established previously [8], [16].

The resulting mate-paired reads were initially mapped to the NCBI reference genome (build 37.1, GRCh37/hg19) using a fast algorithm, and these initial mappings were both expanded and refined by local *de novo* assembly, which was applied to all regions of the genome that appeared to contain variations based on initial mappings [8]. Mapped reads were assembled into a best-fit diploid sequence with two separate resultant sequences for each locus in diploid regions (exceptions: mitochondria were assembled as haploid, and for males the non-pseudo-autosomal regions were assembled as haploid) by using a custom software suite that implements both Bayesian and de Bruijn graph techniques [8]. Variants were called by independently comparing each of the diploid assemblies to the reference. Data for each genome were delivered as lists of sequence variants (SNPs, short indels, and block substitutions) relative to the NCBI reference genome accompanied with variant quality scores. Block substitutions were called where a series of nearby reference bases had been replaced with a different series of bases in an allele. Block substitutions can be length-conserving (the same number of bases as the corresponding reference sequence region) or length-altering. For sites with multiple variant bases, if at least two reference bases on both alleles were called between two variant sequences, then the sites were broken into smaller variant events. The sequence variant data from this study are available at http://tulane.edu/publichealth/bio/genetics-and-genomics-study-of-osteoporosis.cfm.

### CNV analysis

CNVs were identified by using *CNV-seq* (http://tiger.dbs.nus.edu.sg/cnv-seq/) [39], which compares the numbers of mapped reads in a sliding window between two individuals and assesses the hypothesis of no copy number variation based on a probabilistic model. For this analysis, one of the sequenced genomes was randomly picked as the reference genome and the remaining 43 genomes were compared with this reference genome for numbers of mapped reads in 2 kb sliding windows.

### Variant annotation

Based on their locations mapped to the RefSeq transcripts, the identified variants were classified into different groups, including intergenic, CDS, intron, splicing donor and acceptor sites, and 5′- /3′- untranslated regions (UTRs). Since variants may be fall into multiple categories depending on the alternative transcript isoforms examined, a hierarchy CDS>UTRs>splicing sites>intron>intergenic was applied, such that a variant was only counted once at its highest level in the hierarchy. SNPs and indels mapped to CDS and splicing sites were further assessed for their potential effects on the gene products to identify potential LoF variants that are predicted to create or disrupt a stop codon, shift the normal reading frame, and disrupt a splice site. The identification of LoF variants was performed by using the Variant Annotation Tool (version 2.0.1.) [32] with the GENCODE v7 annotation reference [33].

The program *Gorilla* (http://cbl-gorilla.cs.technion.ac.il/) [40] was employed to test whether genes containing high or low density of amino acid changing variants (non-synonymous SNPs and frameshift indels/block substitutions) were enriched in certain GO terms (biological processes). The density of deleterious coding variants for each Refseq gene was calculated by dividing the total number of deleterious variants in the corresponding Refseq transcripts by the sum of CDS lengths of the transcripts. A ranked list of genes was generated by sorting the Refseq genes in three steps, first based on the density of deleterious coding variants, then by the total number of deleterious variants, and finally by the sum of CDS lengths. The ranked list of genes was then uploaded into *Gorilla* for GO enrichment analysis.

Non-synonymous SNPs identified in these personal genomes were screened for known associations with inherited disorders/traits by using *Trait-o-matic* (https://github.com/xwu/trait-o-matic), which finds and cross-references the non-synonymous SNPs with records in the Online Mendelian Inheritance in Man (OMIM) database (http://www.omim.org).

### Variant correlation

The human genome was divided into consecutive 300-kb windows and the numbers of different types of variants (SNP, insertion, deletion, and block substitution) were counted within each window. Pairwise Pearson's correlation coefficients between different types of variants were computed using variant counts from 100 consecutive windows through the *cor* function in R.

### Genome-wide SNP genotyping

Among the 44 subjects, 20 have been genotyped using the Affymetrix Genome-Wide Human SNP Array 6.0 (Affymetrix, Santa Clara, CA, USA) through our recent genome-wide association study for BMD (unpublished data). Genotyping was performed following the manufacturer's recommended protocol. SNPs were identified using Birdsuite (version 1.5.2, http://www.broad.mit.edu/mpg/birdsuite/analysis.html).

## Supporting Information

Figure S1 Genome-wide distribution of SNPs, indels, block substitutions, and proportion of novel SNPs.
(PDF)

Figure S2 Genome-wide correlations between different types of variants.
(PDF)

Figure S3 SNP allele frequency and size distributions of indels and block substitutions.
(PDF)

Figure S4 Size distribution of identified CNVs.
(PDF)

Table S1 Characteristics of study subjects and genome sequencing data.
(XLS)

Table S2 Top 5 regions showing highest proportion of novel SNPs.
(PDF)

Table S3 Fifteen loss-of-function variants that were presented as homozygous form in all 44 genomes.
(PDF)

Table S4 Heterozygous autosomal dominant and homozygous autosomal recessive variants identified in the 44 genomes.
(PDF)

Table S5 Summary of variants identified on mitochondrial and Y chromosome.
(PDF)
