# Supplementary material for: Comprehensive Characterization of Human Genome Variation by High Coverage Whole-Genome Sequencing of Forty Four Caucasians
Source: PLoS One. 2013 Apr 5;8(4):e59494. doi: 10.1371/journal.pone.0059494 (PMC3618277; doi:10.1371/journal.pone.0059494)
Supplement: Figure S1 — Genome-wide distribution of SNPs, indels, block substitutions, and proportion of novel SNPs. (PDF) [file pone.0059494.s001.pdf]

Figure S1. Genome-wide distribution of A) SNPs, B) indels, C) block substitutions, and D) proportion of novel SNPs.

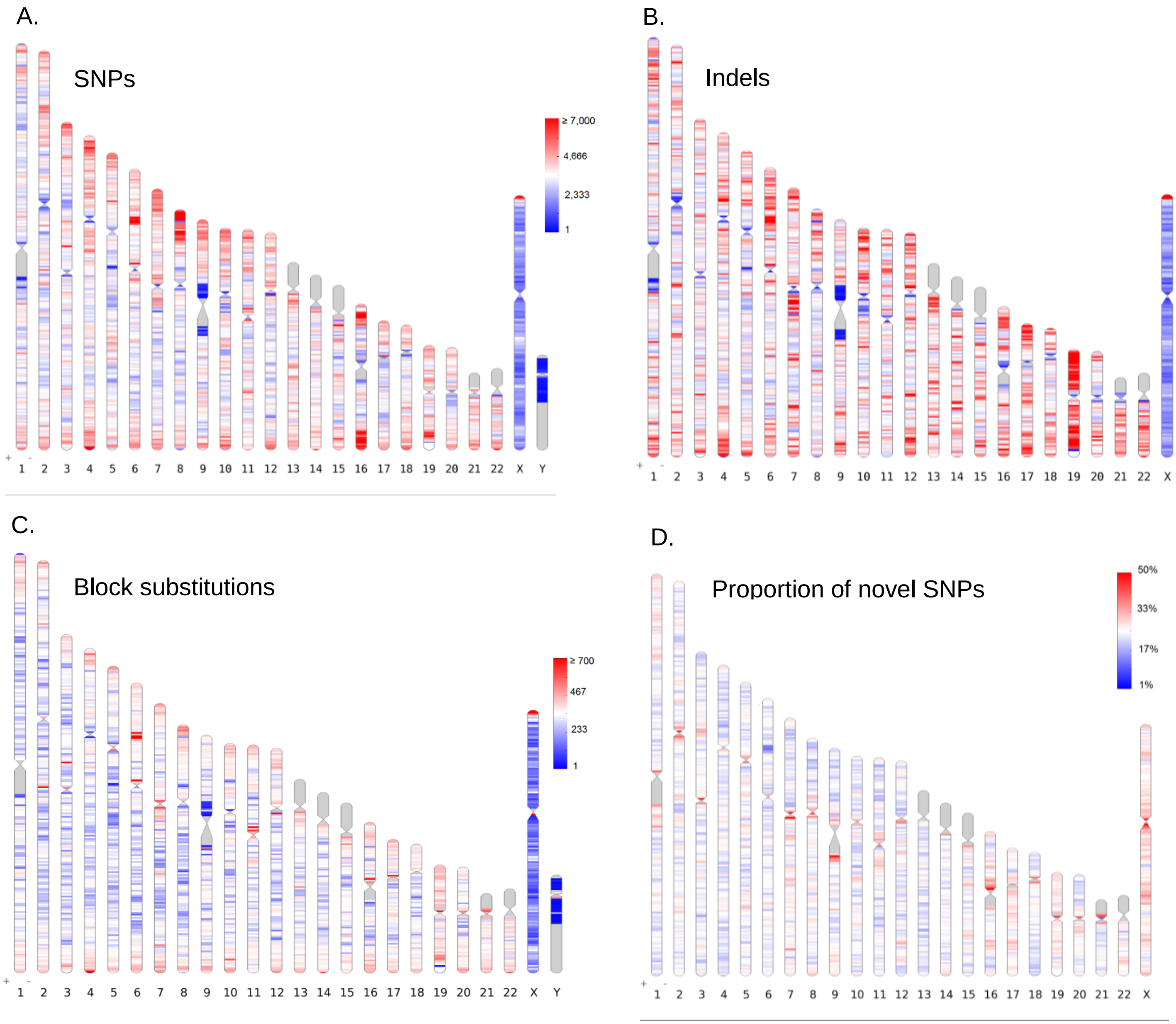

Total number of SNP, indels and block substitutions were calculated in non-overlap 1-megabases (Mb) windows across the human genome. The proportion of novel SNPs in each 1Mb window was calculated as the percentage of all the identified SNPs that were not archived in dbSNP v131 or the 1000 Genomes Project Phase 1 data. Ideograms were plotted using *Idiographica* (Kin and Ono 2007). The diversities were illustrated by colors, with red indicating higher numbers and blue indicating lower numbers. Genomic regions in which no indels or block substitutions were identified or no reference sequences could be determined are shown in grey.
