# Supplementary material for: Comprehensive Characterization of Human Genome Variation by High Coverage Whole-Genome Sequencing of Forty Four Caucasians
Source: PLoS One. 2013 Apr 5;8(4):e59494. doi: 10.1371/journal.pone.0059494 (PMC3618277; doi:10.1371/journal.pone.0059494)
Supplement: Figure S2 — Genome-wide correlations between different types of variants. (PDF) [file pone.0059494.s002.pdf]

Figure S2. Genome-wide correlations between different types of variants. A) SNP~insertions, B) SNP~deletions, C) SNP~block substitutions, D) insertions~deletions, E) insertions~block substitutions, and F) deletions~block substitutions. The genomes were divided into consecutive 300-kb binned regions and counts of different types of variants were obtained for these binned regions. Pearson's correlation coefficient between two types of variants then was computed using variant counts from 100 consecutive binned regions through the *cor* function in R.

A.

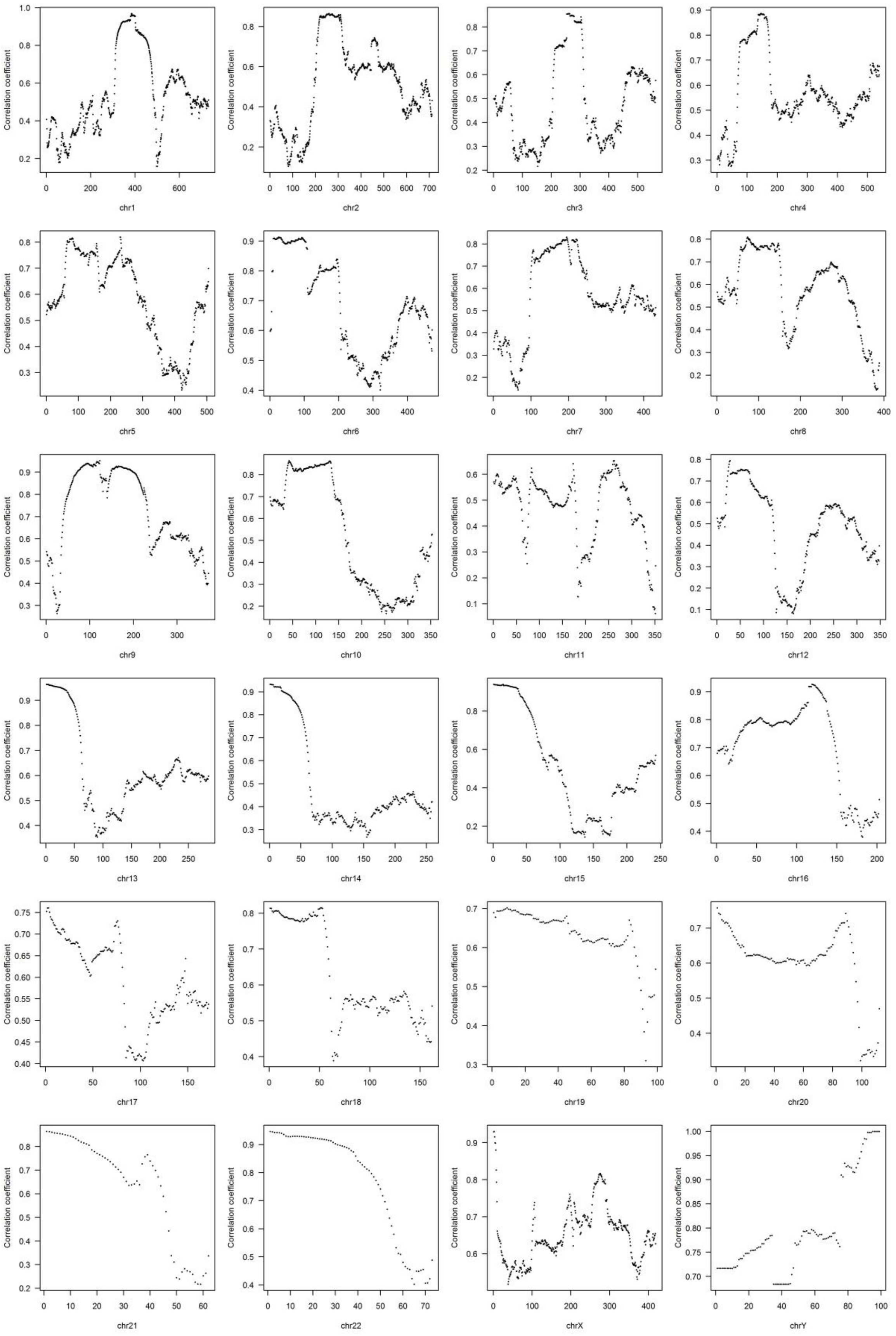

B.

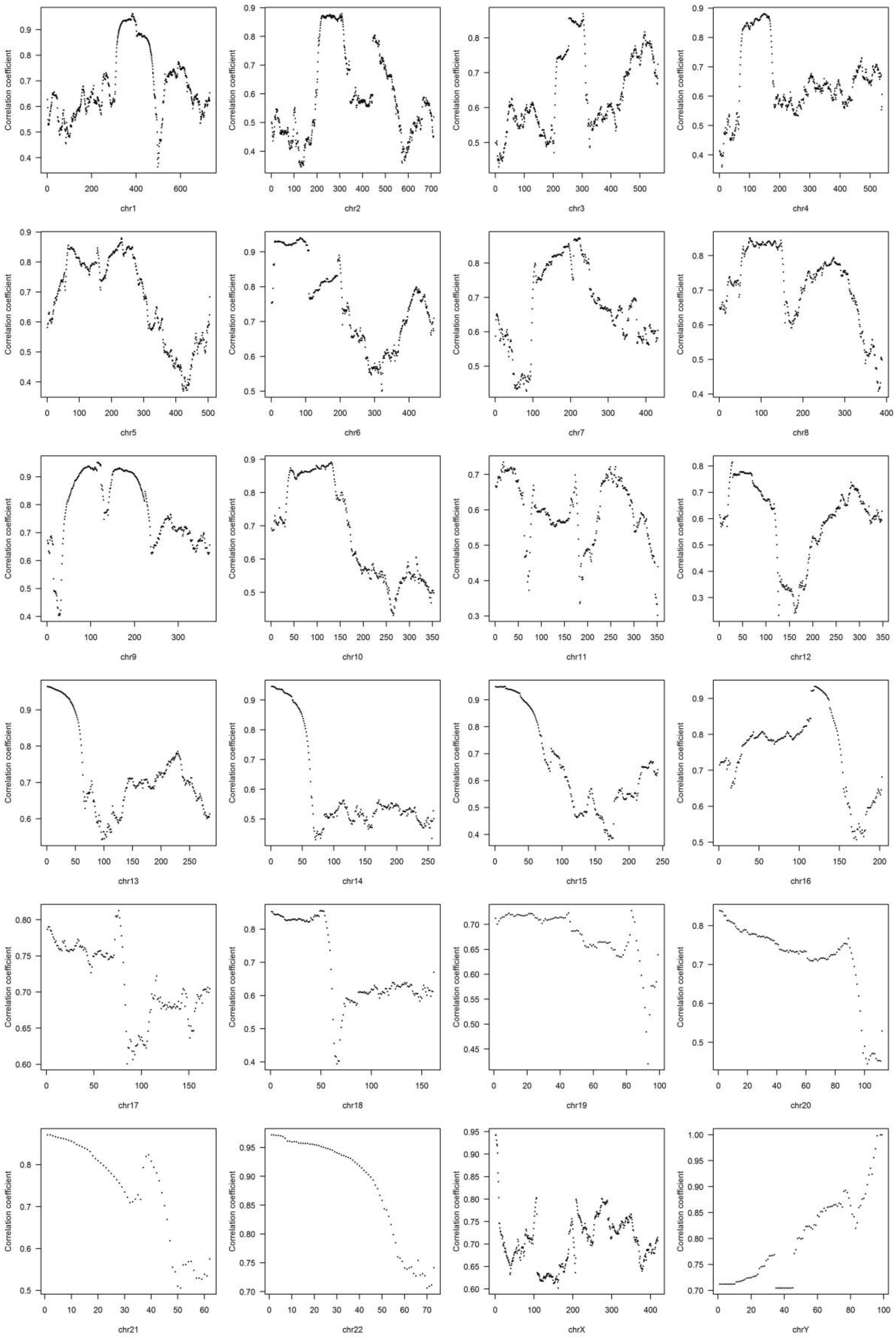
Correlation coefficient
chr1
chr2
chr3
chr4
chr5
chr6
chr7
chr8
chr9
chr10
chr11
chr12
chr13
chr14
chr15
chr16
chr17
chr18
chr19
chr20
chr21
chr22
chrX
chrY

C.

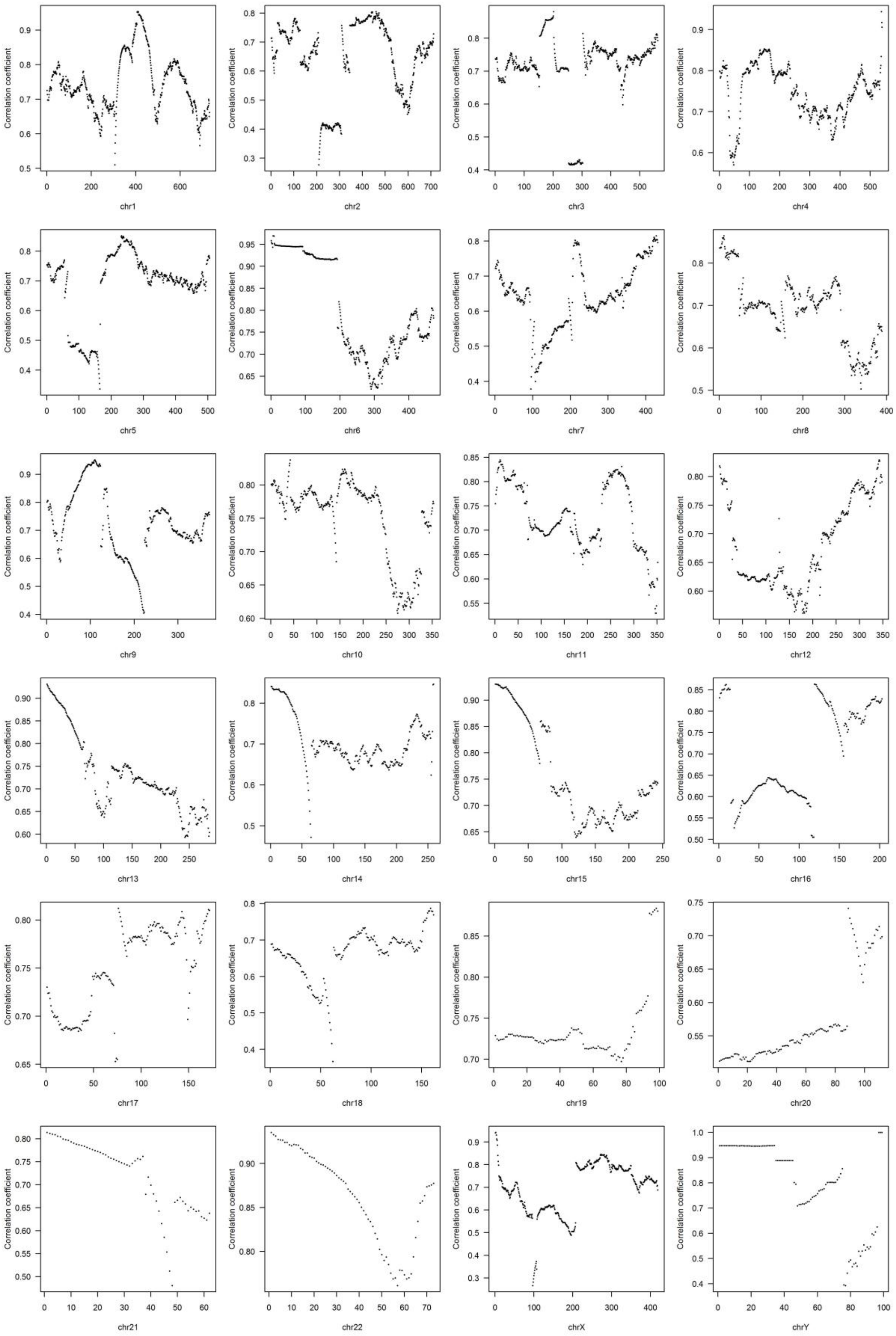
Correlation coefficient
chr1
chr2
chr3
chr4
chr5
chr6
chr7
chr8
chr9
chr10
chr11
chr12
chr13
chr14
chr15
chr16
chr17
chr18
chr19
chr20
chr21
chr22
chrX
chrY

D.

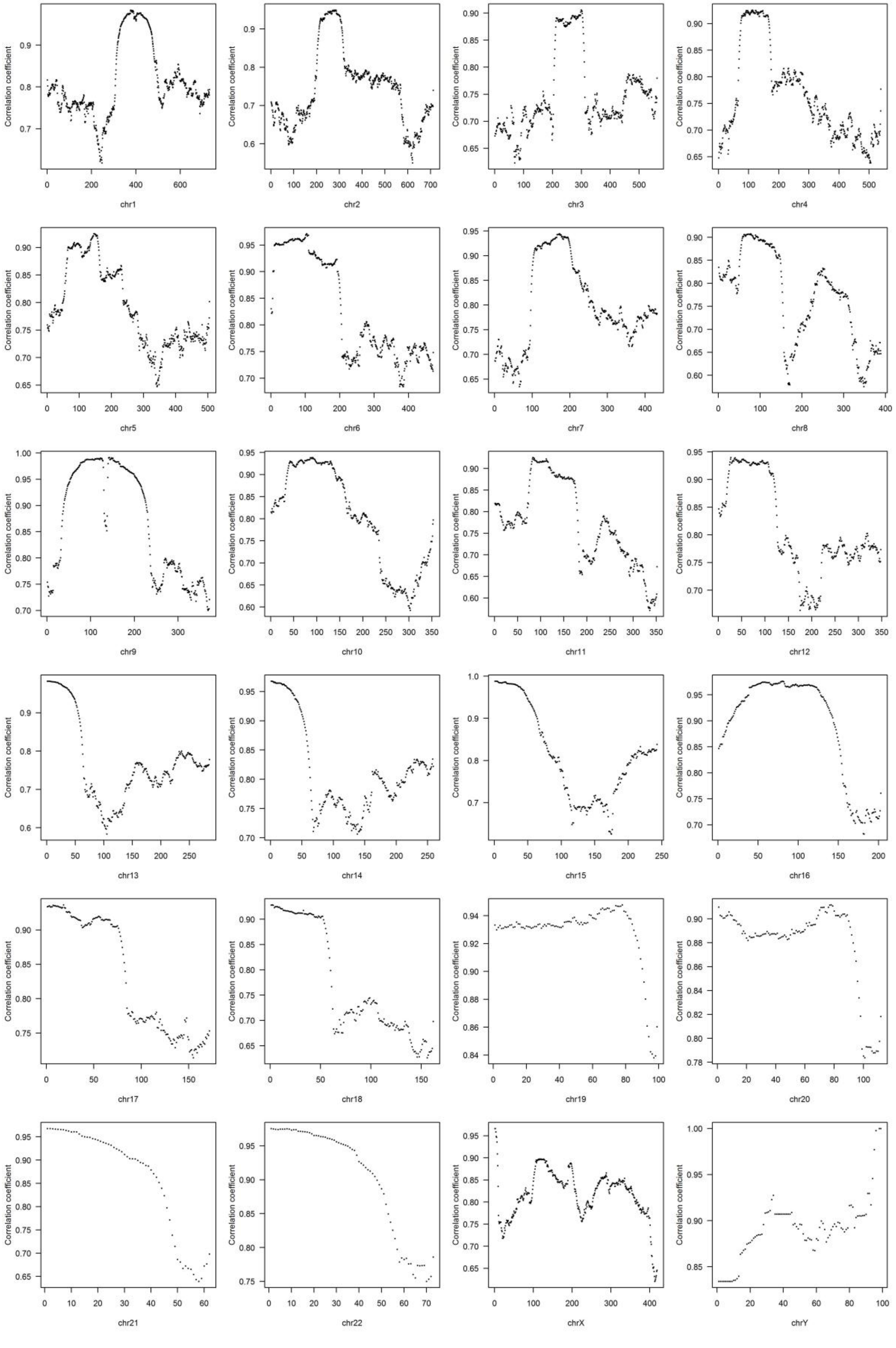

Correlation coefficient
chr1
chr2
chr3
chr4
chr5
chr6
chr7
chr8
chr9
chr10
chr11
chr12
chr13
chr14
chr15
chr16
chr17
chr18
chr19
chr20
chr21
chr22
chrX
chrY

E.

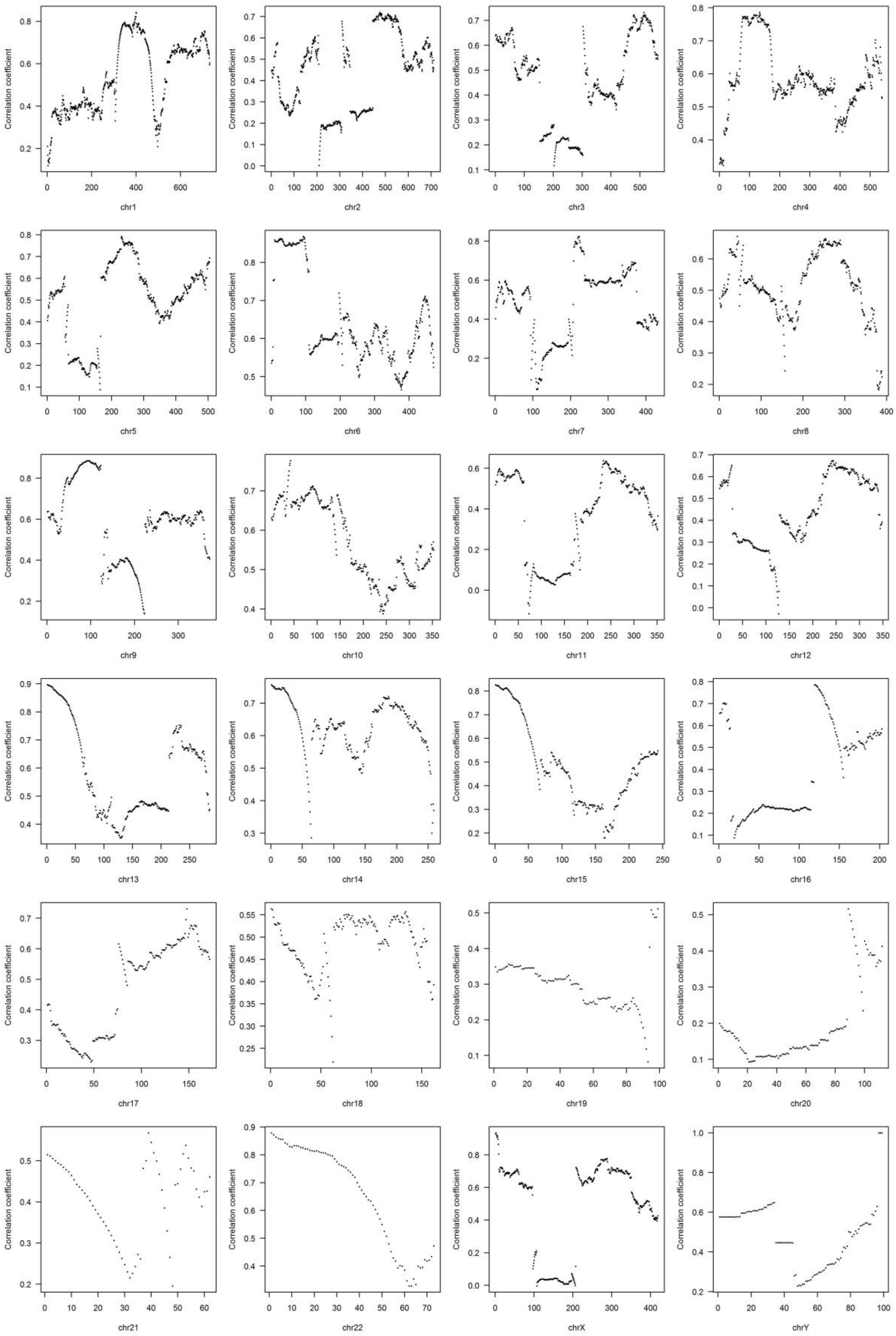
Correlation coefficient
chr1
chr2
chr3
chr4
chr5
chr6
chr7
chr8
chr9
chr10
chr11
chr12
chr13
chr14
chr15
chr16
chr17
chr18
chr19
chr20
chr21
chr22
chrX
chrY

F.

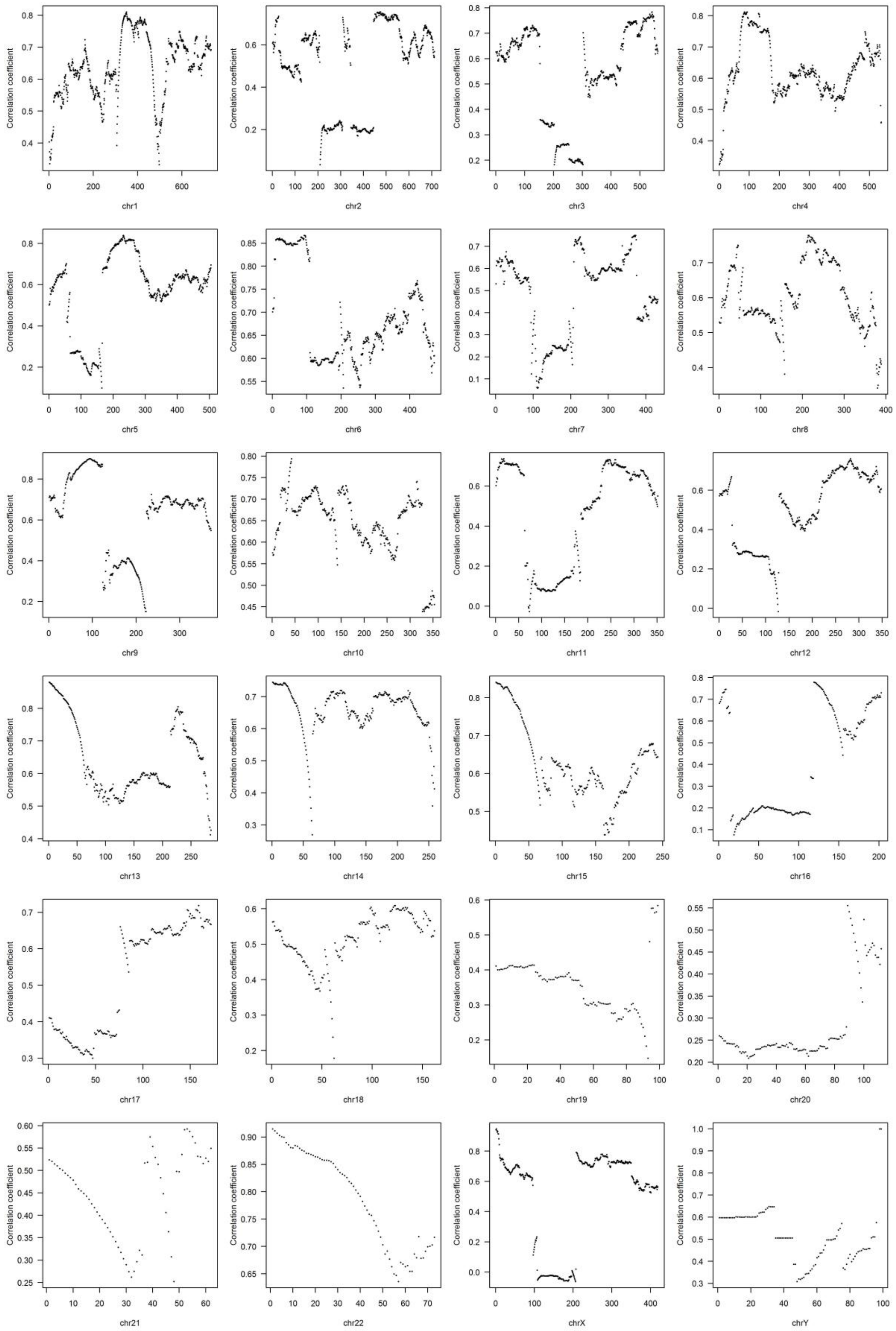
Correlation coefficient
chr1
chr2
chr3
chr4
chr5
chr6
chr7
chr8
chr9
chr10
chr11
chr12
chr13
chr14
chr15
chr16
chr17
chr18
chr19
chr20
chr21
chr22
chrX
chrY
