# Supplementary material for: Comprehensive Characterization of Human Genome Variation by High Coverage Whole-Genome Sequencing of Forty Four Caucasians
Source: PLoS One. 2013 Apr 5;8(4):e59494. doi: 10.1371/journal.pone.0059494 (PMC3618277; doi:10.1371/journal.pone.0059494)

Figure S3. SNP allele frequency and size distributions of indels and block substitutions. **A**, Fraction of nonsynonymous, synonymous, and noncoding SNPs in each minor allele frequency (MAF) class. **B-D**, Size distribution of indels in **B)** coding regions, **C)** introns, and **D)** UTRs, respectively. **E**, Distribution of change in sequence length caused by block substitutions.

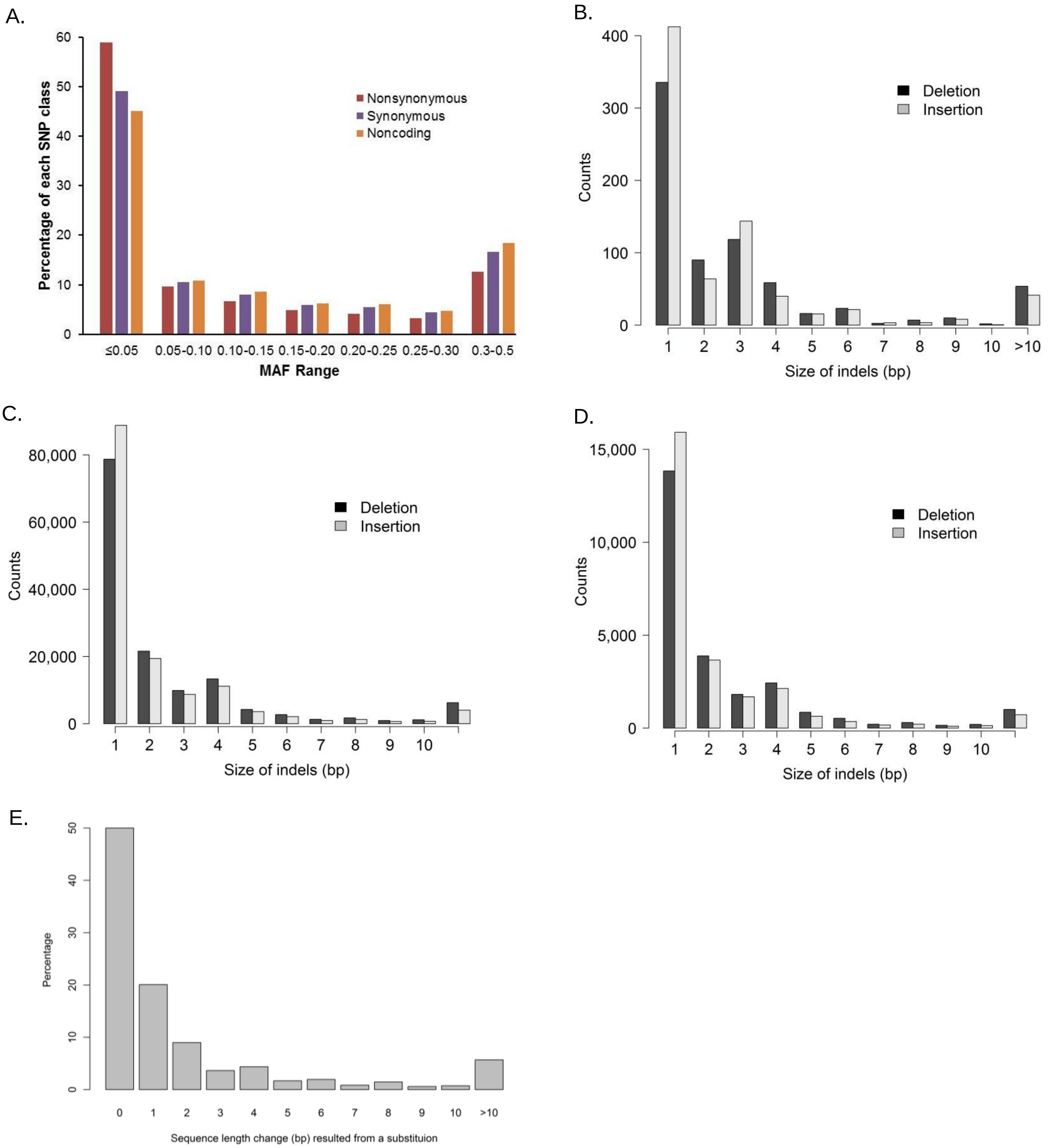

Supplement: Figure S3 — SNP allele frequency and size distributions of indels and block substitutions. (PDF) [file pone.0059494.s003.pdf]
