# Supplementary figures and images for: Comprehensive Characterization of Human Genome Variation by High Coverage Whole-Genome Sequencing of Forty Four Caucasians
Source: PLoS One. 2013 Apr 5;8(4):e59494. doi: 10.1371/journal.pone.0059494 (PMC3618277; doi:10.1371/journal.pone.0059494)

**Figure S4. Size distribution of identified CNVs.**

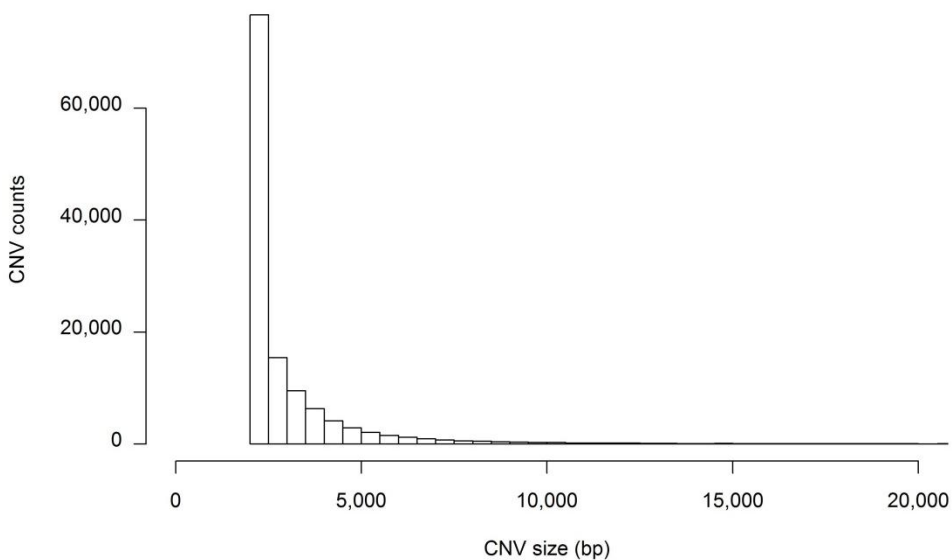

Supplement: Figure S4 — Size distribution of identified CNVs. (PDF) [file pone.0059494.s004.pdf]
