# Supplementary material for: Comprehensive Characterization of Human Genome Variation by High Coverage Whole-Genome Sequencing of Forty Four Caucasians
Source: PLoS One. 2013 Apr 5;8(4):e59494. doi: 10.1371/journal.pone.0059494 (PMC3618277; doi:10.1371/journal.pone.0059494)
Supplement: Table S2 — Top 5 regions showing highest proportion of novel SNPs. (PDF) [file pone.0059494.s006.pdf]

Table S2. Top 5 regions showing highest proportion of novel SNPs

Regions	Positions	Current Study	dbSNP v131	1000 G	Current study vs. dbSNP (Novel rate)	Current Study vs. 1000 G (Novel rate)	1000 G vs. dbSNP (Novel rate)
Xq11.1	61800001-62100000	1814	9123	3625	308 (83%)	245 (86%)	206 (91%)
Xq11.1	61500001-61800000	955	10289	600	235 (75%)	28 (97%)	18 (87%)
16p11.1	35100001-35400000	1104	7931	2146	261 (76%)	162 (85%)	697 (84%)
3p11.1	90300001-90600000	1126	9126	2427	229 (80%)	223 (80%)	714 (87%)
7q11.1-q11.2	61500001-61800000	915	9761	1540	159 (83%)	138 (85%)	1028 (87%)

Numbers shown are total numbers of SNPs identified in the selected 1Mb regions from each data set, and numbers of SNPs shared between two data sets. 1000 G: data from the 1000 Genome Project Phase 1 data released on 5/21/2011.
