# Supplementary material for: Comprehensive Characterization of Human Genome Variation by High Coverage Whole-Genome Sequencing of Forty Four Caucasians
Source: PLoS One. 2013 Apr 5;8(4):e59494. doi: 10.1371/journal.pone.0059494 (PMC3618277; doi:10.1371/journal.pone.0059494)
Supplement: Table S3 — Fifteen loss-of-function variants that were presented as homozygous form in all 44 genomes. (PDF) [file pone.0059494.s007.pdf]

Table S3. Fifteen loss-of-function variants that were presented as homozygous form in all 44 genomes

Position	varType	dbSNP	Ref	Calls	Impact	Gene
chr1:156565050-156565050	ins	rs10637036	-	CA	Frameshift	GPATCH4
chr11:46342261-46342261	ins	rs71038900	-	G	Frameshift	CREB3L1
chr11:125452303-125452303	ins	rs55912941	-	C	Frameshift	EI24
chr16:138773-138773	ins	rs57321480	-	G	Frameshift	C16orf35
chr17:61660894-61660895	del	rs67652163	G	-	Frameshift	DCAF7
chr19:36214633-36214633	ins	rs11373774	-	G	Frameshift	MLL4
chr19:58718361-58718361	ins	rs59557917	-	G	Frameshift	ZNF274
chr22:19189004-19189004	ins	rs11386977	-	C	Frameshift	CLTCL1

"Ref" represents the sequences at the corresponding positions in the NCBI human reference genome (build 37.1, GRCh37/hg19), and "Calls" represents sequences called in our samples.
