# Supplementary material for: Comprehensive Characterization of Human Genome Variation by High Coverage Whole-Genome Sequencing of Forty Four Caucasians
Source: PLoS One. 2013 Apr 5;8(4):e59494. doi: 10.1371/journal.pone.0059494 (PMC3618277; doi:10.1371/journal.pone.0059494)
Supplement: Table S4 — Heterozygous autosomal dominant and homozygous autosomal recessive variants identified in the 44 genomes. (PDF) [file pone.0059494.s008.pdf]

Table S4. Heterozygous autosomal dominant and homozygous autosomal recessive variants identified in the 44 genomes

Chr.	Position	AA change	Gene	OMIM accession	Phenotype	# of subjects	MAF
Heterozygous Autosomal Dominant Variants							
1	12064892	R468H	MFN2	608507.0015	Charcot-Marie-Tooth disease-2A2	2	NA
1	147380823	I247M	GJA8	600897.0003	zonular pulverulent cataract	1	NA
2	71058878	W264R	CD207	604862.0001	Birbeck granule deficiency	2	NA
2	216235046	Q1974R	FN1	135600.0002	glomerulopathy with fibronectin deposits 2	1	NA
4	88533540	R68W	DSPP	125485.0006	type II dentinogenesis imperfecta	2	NA
7	19156382	S188L	TWIST1	601622.0014	craniosynostosis type 1	1	NA
10	31810782	Q840P	ZEB1	189909.0004	Fuchs endothelial corneal dystrophy-6	1	NA
10	88446830	D117N	LDB3	605906.0007	dilated cardiomyopathy with left ventricular noncompaction	1	NA
12	57431402	G662E	MYO1A	601478.0005	autosomal dominant nonsyndromic deafness-48	7	0.025
12	122295335	A33T	HPD	609695.0005	hawkinsinuria	11	0.125
15	48779352	R1170H	FBN1	134797.0032	subdiagnostic variant of Marfan syndrome	1	NA
17	16852187	C104R	TNFRSF13B	604907.0001	common variable immunodeficiency-2 / immunoglobulin A (IgA) deficiency-2	2	0.014
17	72745313	L110V	SLC9A3R1	604990.0001	hypophosphatemic nephrolithiasis/osteoporosis-2	3	0.034
21	47552201	P932L	COL6A2	120240.0010	Bethlem myopathy	1	NA
Homozygous Autosomal Recessive Variants							
1	158624528	A970D	SPTA1	182860.0009	hereditary spherocytosis-3	1	0.042
10	13340236	P29S	PHYH	602026.0006	adult Refsum disease	1	0.150
16	48258198	G180R	ABCC11	607040.0001	wet/dry ear wax	1	0.125
20	43255220	K80R	ADA	608958.0001	severe combined immunodeficiency, due to adenosine deaminase deficiency	1	0.033

“AA change”: amino acid changes by the variants. “MAF”: minor allele frequency reported in dbSNP.
