# Supplementary material for: Comprehensive Characterization of Human Genome Variation by High Coverage Whole-Genome Sequencing of Forty Four Caucasians
Source: PLoS One. 2013 Apr 5;8(4):e59494. doi: 10.1371/journal.pone.0059494 (PMC3618277; doi:10.1371/journal.pone.0059494)
Supplement: Table S5 — Summary of variants identified on mitochondrial and Y chromosome. (PDF) [file pone.0059494.s009.pdf]

Table S5. Summary of variants identified on mitochondrial and Y chromosome

**Variant Type**	**No. of SNPs (% novel)**	**No. of Indels and block substitutions (% of novel)**
Mitochondria	285 (53.7%)	21 (90.5%)
Noncoding	90 (54.4%)	20 (90.0%)
Coding	195 (53.3%)	1 (100%)
Y Chromosome	8,673 (54.3%)	2,348 (65.2%)
Intergenic	7,915 (54.0%)	2,170 (63.8%)
Intragenic	758 (57.5%)	178 (82.3%)
Intron	578 (62.6%)	144 (84.0%)
UTR	139 (43.9%)	28 (82.1%)
Splicing acceptor site	1 (0%)	1 (100%)
Splicing donor site	0	0
Coding domain	40 (32.5%)	5 (40.0%)
